# Analysis of MHC class I and II expression in relation to presence of HPV genotypes in premalignant and malignant cervical lesions.

**DOI:** 10.1038/bjc.1993.254

**Published:** 1993-06

**Authors:** F. V. Cromme, C. J. Meijer, P. J. Snijders, A. Uyterlinde, P. Kenemans, T. Helmerhorst, P. L. Stern, A. J. van den Brule, J. M. Walboomers

**Affiliations:** Institute for Pathology, Free University Hospital, Amsterdam, The Netherlands.

## Abstract

**Images:**


*Br. J. Cancer* (1993), **67**, 1372–1380 © Macmillan Press Ltd., 1993

### Analysis of MHC class I and II expression in relation to presence of HPV genotypes in premalignant and malignant cervical lesions

F.V. Cromme[1], C.J.L.M. Meijer[1], P.J.F. Snijders[1], A. Uyterlinde[1], P. Kenemans[2], Th. Helmerhorst[2], P.L. Stern[3], A.J.C. van den Brule[1] & J.M.M. Walboomers[1]

[1]*Institute for Pathology, Section of Molecular Pathology;* [2]*Department of Gynaecology, Free University Hospital, De Boelelaan 1117, 1081 HV Amsterdam, The Netherlands;* [3]*Paterson Institute for Cancer Research, Christie Hospital (NHS) Trust, Wilmslow Road, Manchester, M20 9BX, UK.*

**Summary** Cervical intraepithelial neoplasia (CIN) grades I to III lesions ($n = 94$) and squamous cell carcinomas of the uterine cervix ($n = 27$) were analysed for MHC class I and II expression and presence of HPV genotypes.

MHC class I and II expression was studied by immunohistochemistry and HPV typing was performed by general primer- and type-specific primer mediated PCR (GP/TS PCR). Both techniques were performed on paraffin embedded tissue sections.

Results show disturbed MHC class I heavy chain expression in CIN I to CIN III, as well as in cervical carcinomas. Upregulated MHC class II expression on dysplastic epithelial cells was also found in the different CIN groups and carcinomas. Prevalence of HPV genotypes increased with the severity of the lesion, mainly due to the contribution of the HPV types 16 and 18. No correlation could be established between the presence of specific HPV genotypes and any MHC expression pattern in the different CIN groups or cervical carcinomas. In some cases these data were confirmed by RNA *in situ* hybridisation showing HPV 16 E7 transcripts in the same dysplastic/neoplastic cells from which MHC status was determined. The results indicate that local differences may exist in the type of cellular immune response to HPV induced lesions.

A central role in the antigen-specific immune response is played by the major histocompatibility complex (MHC), which are cell surface proteins that act as restricting elements in the recognition of antigen by T-cells. MHC class I (MHC-I) present endogenous antigen to cytotoxic T-lymphocytes (CTLs). Low levels or lack of MHC-I surface expression can consequently render aberrant cells non-immunogenic to CTLs, and may provide a way for cells to escape immune surveillance. MHC-I alterations have been described in human cancer of different sites of the body (see review Ruiz-Cabello *et al.*, 1991).

Generally, MHC class II (MHC-II) surface expression is restricted to specialised antigen presenting cells (APCs), that present mainly opsonised exogenous antigen to T-helper cells. Recognition leads to activated T-cells, which can stimulate B-cell, CTL proliferation and MHC non-restricted killing by natural killer (NK) cells or activated macrophages. Occasionally, other cells like neoplastic epithelial cells have been described to express MHC-II, which could assist in the onset of the cellular immune response (Ostrand-Rosenberg *et al.*, 1991).

Infections with specific human papillomavirus (HPV) types are strongly associated with the development of cervical cancer, with HPV types 16 and 18 as the most predominant types (Zur Hausen, 1989). This is supported by the increasing prevalence of HPV 16/18 with increasing severity of dysplasia to cervical carcinoma (van den Brule *et al.*, 1991; Lungu *et al.*, 1992) and the capacity of these viruses to transform *in vitro* their natural host cells, the keratinocytes. These cells are immortalised (Dürst *et al.*, 1987; Pirisi *et al.*, 1987) and show abnormal differentiation (McCance *et al.*, 1988; Woodworth *et al.*, 1990), but are initially non-tumorigenic in animals. This indicates that additional events besides infections with high risk HPVs are involved in the pathogenesis of cervical cancer. These events may include activation of cellular proto-oncogenes, genetic inactivation of tumour suppressor genes, HPV integration into the cellular genome and failure of immune surveillance (Zur Hausen, 1989*a*). In accordance with the last fact it has been shown recently that altered expression of MHC-I and -II is present in a substantial number of HPV 16/18 DNA containing cervical carcinomas (Connor & Stern, 1990; Glew *et al.*, 1992), suggesting that changes in the presentation of viral tumour antigens to the cellular immune system can occur. However, to get insight whether altered MHC-I and -II expression is related to the development of cervical cancer from its premalignant lesions, it is necessary to study dysplastic cervical lesions (CIN) for the MHC-I and -II status.

Tumour virus based mechanisms have been described that specifically influence MHC-I cell surface expression (Signas *et al.*, 1982; Schrier *et al.*, 1983). Similar mechanisms could exist for HPV affecting antigen presentation of the infected cells. However, little is known about MHC alterations in CIN lesions in relation to the presence of different HPV genotypes.

Therefore in this study expression of MHC-I and -II was investigated in CIN lesions of different grades and cervical carcinomas. HPV typing was carried out by a combined general primer-mediated (GP-) and type-specific (TS-) polymerase chain reaction (PCR) strategy (van den Brule *et al.*, 1991; Walboomers *et al.*, 1992). In addition, HPV RNA *in situ* hybridisation (RISH) was applied to some HPV 16 PCR positive lesions, in order to localise cells containing transcriptionally active HPV 16 in relation to altered MHC expression. The results indicate that MHC-I and -II alterations are also present in CIN lesions, independent from the presence of HPV DNA.

### Materials and methods

### *Tissues*

Tissues were obtained from patients attending the oncological gynaecological outpatient department from the Free University Hospital, Amsterdam, for routine diagnostic and therapeutic procedures. Clinical material ranged from colposcopically directed punch biopsies to resection specimens. Formalin fixed, paraffin embedded tissues were cut to 4 $\mu m$ thick consecutive sections for MHC expression analysis by immunohistochemistry and for PCR analysis, sandwiched by two sections for haematoxylin-eosin (HE) staining. CIN lesions were histologically reviewed by two pathologists on both HE

Correspondence: J.M.M. Walboomers, Institute for Pathology, Section of Molecular Pathology, Free University Hospital, De Boelelaan 1117, 1081 HV Amsterdam, The Netherlands.
Received 22 October 1992; and in revised form 14 January 1993.

stained sections. In case of disagreement a consensus diagnosis was reached. CIN lesions were classified as grade I, II or III, depending on the thickness of the epithelium involved by dysplastic cells. Thus grade 1 CIN represents less than one third involvement of the thickness of the epithelium, grade II one third to two third, and grade III two third to full thickness (Richart, 1987).

*Immunohistochemistry*

Paraffin embedded sections on coated slides (0.1% Poly-L-lysine) were deparaffinised with xylene, rehydrated and endogenous peroxidase was blocked by incubating 30 min with methanol, containing 0.3% $H_2O_2$. After rinsing in PBS, sections were treated as follows for the different primary antibodies: for Pan keratin sections were incubated with trypsin (0.5% w/v) in 0.5% $CaCl_2$ (pH 7.8) for 30 min at 37°; for RaHC sections were treated with 0.2 M NaAc (pH 4.8) for 15 min at RT; for LN3 sections were treated with a saturated solution of lead thiocyanate for 2 × 5 min at 100°C. After washing repeatedly in PBS, sections were preincubated with normal goat (1:20) or horse (1:50) serum, depending on the secondary antibodies used, for 20 min and incubated with specific antibodies. The following primary antibodies were incubated at RT for 30 min: polyclonal RaHC, 1:500, specific for HLA-A, -B and -C heavy chains (Stam *et al.*, 1986); monoclonal antibody (MoAb) HC10, 1:800, preferentially recognising HLA-B and -C locus products (Stam *et al.*, 1986); Polyclonal Pan Keratin, 1:400, recognising a broad spectrum of cytokeratins (Dako Patts, Glostrup, Denmark). MoAb LN3, 1:25, recognising HLA-DR antigens (Biotech, Breieich, FRG) was incubated overnight at 4°C.

Monoclonal antibodies (LN3, HC10) were detected by a biotinylated horse-anti mouse Ab 1:500 (Vector Lab, Burlingame, USA), polyclonals (RaHC, Polyclonal Keratin) with biotinylated goat anti-rabbit 1:500 (Vector Lab). All secondary antibodies were diluted in PBS, containing 2% (v/v) human serum and 1% (w/v) bovine serum albumin (Sigma, Deisenhofen, FRG). Detection was performed using horse radish peroxidase coupled to avidin-biotin complex (Vector Elite, Vector Lab), after which the complex was visualised using diaminobenzidine and $H_2O_2$. Slides were counterstained with haematoxylin, dehydrated and mounted in Depex.

Immunohistochemical staining was analysed independently by two observers. In case of disagreement staining results were re-analysed by the observers together. The percentage of dysplastic or neoplastic cells that show staining for MHC-I or -II was estimated, with normal epithelium and immunocompetent cells serving as positive internal control for MHC-I and endothelial cells and infiltrating immunocompetent cells for MHC-II. The lesions were classified according to the percentage of dysplastic cells that show positive or strongly reduced to negative staining. For MHC-I expression lesions were classifed into three categories: normal, when 75% or more of the dysplastic cells were positively stained; heterogenous, when 25% to 75% of the dysplastic cells showed a strongly reduced to negative staining; disturbed, when 75% or more of the dysplastic cells showed strongly reduced to negative staining. For MHC-II expression, lesions were classified as altered, when 25% or more of the dysplastic cells showed positive staining for MHC-II. When less than 25% of the dysplastic cells showed positive staining, lesions were categorised as normal.

*Polymerase Chain Reaction (PCR)*

Presence of HPV genotypes was analysed on tissue sections from CIN lesions and cervical carcinomas. For HPV PCR analysis (on tissue specimens) five 4 μm thick formalin fixed, paraffin embedded sections from each sample were deparaffinised with xylene and washed twice with 96% ethanol. After the tissue was centrifuged (1000 *g*, 10 min) and air dried, the pellet was suspended in 50 μl distilled water and frozen at − 80°C for at least 30 min. After thawing a 50 μl proteinase K mix (1.5 mM $MgCl_2$, 0.45% Tween 20 and 60 μg $ml^{-1}$ proteinase K (Boehringer Mannheim, Mannheim, FRG) was added and the mixture was incubated at 55°C for several hours. Samples were then treated at 100°C for 10 min and centrifuged. Twenty μl of the supernatant was used in the PCR reaction. The PCR was performed with general primers (GP 5/6), recognising both sequenced and unsequenced HPV genotypes (Snijders *et al.*, 1990). HPV DNA positive samples were then subjected to type-specific PCR, performed with primers specific for HPV 6, 11, 16, 18, 31 and 33 (van den Brule *et al.*, 1990). Samples positive in the general primer mediated PCR which were negative by type-specific PCR were classified as unsequenced or unidentified HPV genotypes (named HPV X). Cells from SiHa and Hela cell lines, which contain HPV 16 and 18 DNA respectively, served as positive controls for the HPV PCR. Amplification products were analysed by 1.5% agarose gel electrophoresis and Southern blot analysis using HPV-specific probes. Nucleotide sequences of primers and probes used in the PCR, as well as the conditions were published elsewhere (Van den Brule *et al.*, 1990; Walboomers *et al.*, 1992). As a negative control liver sections were cut in between the different specimens and also subjected to PCR analysis. To analyse the quality of target DNA for PCR purposes samples were analysed by PCR using human β-globin gene specific primers. Only β-globin PCR positive samples were included in the study.

*RNA* in situ *hybridisation*

RNA *in situ* hybridisation was performed non-radioactively on 4 μm thick tissue sections as previously described by Oudejans *et al.* (1989). Briefly, tissue sections on coated (0.1% poly-L-lysine) slides were air dried overnight at 37°C and deparaffinised by overnight incubation in xylol at 50°C. After progressive rehydration, sections were treated with Lugol's iodine and sodium thiosulphate, washed in phosphate-buffered saline, pH 7.4 (PBS), and treated subsequently with proteinase K (0.1–1 μg $ml^{-1}$ in 50 mM Tris HCl, pH 7.6, 5 mM EDTA) for 15 min at 37°C. After washing in PBS, sections were postfixed in 4% (w/v) phosphate buffered paraformaldehyde, pH 7.4, for 10 min at room temperature, and washed for 5 min in PBS. Hybridisation mixture, containing 50% (v/v) deionised formamide, 2 × SSC (1 × SSC = 0.15 M NaCl, 0.015 M Na-citrate), 10% (w/v) dextran sulphate, yeast tRNA (50 μg $ml^{-1}$) (Boehringer Mannheim) and saturation levels of biotinylated probe, were applied to the sections followed by heating to 65°–70°C for 7 min. Hybridisation was allowed to occur for 2 h at 50°C. After hybridisation, sections were washed in 0.1 × SSC at 68°C for 60 min, and non-specifically bound RNA-probe was removed using 0.5–1 mg $ml^{-1}$ RNAse A (Boehringer Mannheim) in 10 mM Tris HCl, pH 8.2, 1 mM EDTA and 0.5 M NaCl for 15 min at 37°C. Sections were washed once again in 0.1 × SSC for 30 min at 68°C.

The HPV-16 E7-specific probe (clone 72 kindly provided by Dr H. Smits, Department of Virology, University of Amsterdam) is directed against nt 622–879. An actin probe was used as positive control probe, derived from a human β-actin cDNA (Clontech Lab., Palo Alto, USA) and is specific for the 5′ part of the mRNA. Construction of both probes has been described elsewhere (van den Brule *et al.*, 1991*a*). Biotinylated RNA transcripts, both sense and antisense orientations, were generated using biotin-11 UTP (Sigma) and SP6 or T7 RNA polymerase, following manufacturer's instructions (Promega, Madison, USA). RNA-probes were precipitated with ethanol, pellets were dissolved in distilled water containing 400 units $ml^{-1}$ RNaseIn (Promega), diluted in 500 μl deionised formamide containing 50 μg $ml^{-1}$ yeast tRNA (Boehringer Mannheim) and stored at − 80°C.

Integrity and concentration of probes were determined by Northern blot analysis of serial dilutions of both sense and antisense probes. RNAs were visualised using horse radish peroxidase coupled to avidin-biotin complex (Vector Lab) and diaminobenzidine.

RNA-RNA hybrids were detected immunohistochemically by successive incubations with 1:500 dilution of rabbit anti-biotin immunoglobulin (Enzo Biochemicals, Farmingdale, USA), 1:250 dilution of biotin-labelled goat anti-rabbit antibody (Vector Lab), both 30 min at room temperature and 1:100 dilution of colloidal gold labelled streptavidin (5 nm) (Amersham, Buckinghamshire, UK) for 60 min at room temperature. Sections were postfixed in phosphate buffered glutaraldehyde (2%) for 10 min at room temperature and, to remove chloride ions, washed extensively in water before silver enhancement. Enhancement by the silver lactate/hydroquinone method was performed according to the manufacturer's instructions (IntenSeM system, Amersham), incubating three times for 4 min each at room temperature. Finally, sections were counterstained with haematoxylin, dehydrated and mounted in Depex.

RISH results were analysed using a type II confocal laser-scan microscope with reflex contrast (Zeiss, Oberkochen, FRG).

*Statistical analysis*

Statistical analysis was peformed with a chi-square test using a BMDP statistical software program (Cork, Ireland).

### Results

*MHC class I and II expression*

General antigen conservation in the sections was ascertained with a polyclonal keratin antibody, recognizing a broad spectrum of cytokeratins. All specimens used in this study showed positive staining with the pan keratin antibody. MHC-I heavy chain expression was analysed using two antibodies, polyclonal RaHC and monoclonal HC10. In general, staining with HC10 showed more clearly cell surface localisation of the MHC-I complex on keratinocytes. However, no cases were found that showed different results in MHC-I expression with RaHC and HC10.

Normal epithelium, where present in the section, showed staining for MHC-I on cells at the basal side of the epithelial layer, as well as stromal and immunocompetent cells (Figure 1a). Typical examples of dysplastic cells with positive and disturbed expression of MHC-I are shown in Figures 1b and 1c,d respectively. Disturbance of MHC-I expression was predominantly observed at the basal side of the dysplastic epithelium, while sometimes superficial cells still showed positive staining (Figure 1d). In Figure 1e and f an MHC-I negative and a positive cervical carcinoma is shown, respectively.

The number of lesions, scored as disturbed for MHC-I expression, i.e. 75% or more of the dysplastic cells show strongly reduced to negative staining, was 11 out of 34 CIN I, 10 out of 32 CIN II and 12 out of 28 CIN III lesions. In carcinomas 13 out of 27 cases were scored as disturbed. Heterogeneity of MHC-I expression, i.e. 25 to 75% of the dysplastic cells show strongly reduced to negative staining, was scored in 12 out of 34 CIN I, 15 out of 32 CIN II and 12 out of 28 CIN III lesions. In carcinomas 9 out of 27 cases were scored as heterogenous.

MHC-II expression was determined using LN3, staining HLA-DR locus products. In normal epithelium only a few immunocompetent cells are stained (Figure 2a). Examples of negative (Figure 2b) and upregulated (Figure 2c,d) expression of MHC-II on dysplastic epithelial cells are shown. Cell surface localisation of the MHC-II antigens could sometimes be observed (Figure 2d), although other lesions showed a more diffuse cytoplasmic staining for MHC-II (Figure 2c). The expression pattern of MHC-II in CIN lesions was different from MHC-I. Altered class II expression could occasionally be observed on superficial cells, while basal and parabasal cells did not show upregulated MHC-II expression (Figure 2c). In Figure 2e and f carcinomas with no and upregulated MHC-II expression are shown, respectively.

The number of lesions, scored as altered for MHC-II, i.e. 25% or more of the dysplastic cells show staining, was 12 out of 34 CIN I, 20 out of 32 CIN II and 17 out of 28 CIN III lesions. In carcinomas 14 out of 24 were scored for altered MHC-II expression.

MHC-I and -II expression results per CIN group and for the carcinomas are summarised in Table I. Statistical analysis of these data revealed no significant differences ($P$-value $> 0.01$) in MHC alterations between the different CIN grades and carcinomas.

Taking the CIN lesions and carcinomas together 48 out of 121 samples showed simultaneous changes of MHC-I and -II expression. An additional 59 samples showed changes of one class while the other was normal. The remaining 14 samples showed normal expression of both classes. Since this distribution is not statistically significant ($P > 0.01$) it is concluded that there is no correlation between changes in MHC-I and -II expression.

*HPV genotypes in CIN lesions and cervical carcinomas*

HPV prevalence rates in the different CIN grades and carcinomas are illustrated in Figure 3. An increase of HPV prevalence was associated with an increase of the severity of the lesion. In CIN I, CIN II and CIN III/cervical carcinomas an HPV prevalence of 68%, 91% and 100% was found, respectively. Also the contribution of the HPV types associated with cervical carcinoma (type 16, 18 and 31) increased from 53% in CIN I, 75% in CIN II to 96% in CIN III and 100% in cervical carcinomas. Other HPV types, i.e. HPV 6 and HPV X, were found only in CIN I and II ($n = 11$), whereas in none of the samples HPV 11 or 33 could be detected.

*Association between presence of HPV genotype and MHC alterations*

All three MHC-I expression patterns (i.e. normal, heterogenous and altered) were observed in lesions, positive for 'high-risk' HPV types 16, 18 or 31. Also in lesions, positive for HPV 6, X or negative for any HPV genotype, all three MHC patterns could be found. Using statistical analysis no significant differences in MHC-I expression patterns could be established between the different HPV containing and the HPV negative lesions ($P$-value$> 0.01$).

Both normal and upregulated MHC-II expression was observed on dysplastic cells of lesions, positive for 'high-risk' HPV types, as well as in lesions, positive for HPV 6, X or negative for any HPV genotype. No significant differences in MHC-II expression could be established by statistical analysis between the different HPV containing and HPV negative lesions ($P$-value$> 0.01$). The results are summarised in Table II.

To find out whether HPV, if present, is localised in the same dysplastic cells from which MHC status was determined, additional RNA *in situ* hybridisation (RISH) was performed on consecutive sections, using an E7-specific RNA probe. Several PCR HPV 16 DNA positive CIN lesions and cervical carcinomas were analysed with RISH, and two typical examples of CIN lesions are shown in Figure 4. An MHC-I positive CIN II lesion is shown with membranous staining of dysplastic cells at the basal side of the epithelial layer (Figure 4a). RISH reveals presence of HPV 16-E7 transcripts in the same area, with signals restricted to the dysplastic cells (Figure 4b). In Figure 4c and d a detail of a CIN I lesion is shown with heterogenous staining for MHC-I heavy chains in the dysplastic cells. Also in this dysplastic area HPV 16-E7 mRNAs can be detected (Figure 4d). The E7 sense RNA-probe was used as a negative control and gave no signal (not shown). Also in nine carcinomas, described earlier (van de Brule *et al.*, 1991*a*), HPV 16 E7 transcripts were found in all carcinoma cells. In these samples both normal and altered MHC-I and -II expression could be detected.

**Figure 1** MHC class I expression (MHC-I) in normal epithelium **a**, CIN lesions **b-d**, and cervical carcinomas **e**, **f**, as shown by MoAb HC10, directed against HLA-B and -C locus products. Size bars represent 50 μm. **a**, Normal squamous epithelium of the uterine cervix, showing staining of basal and parabasal cells. **b**, Grade III CIN lesion, showing staining of all atypical cells in the full thickness of the epithelium. **c**, CIN II lesion showing staining of the lower half of the surface epithelium. Note that MHC-I expression in the atypical metaplastic cells of the endocervical tube is absent. **d**, CIN III lesion with only the upper third of the epithelium staining for MHC-I. Note the staining of the mononuclear cells in the stroma. **e** and **f**, representative cervical carcinomas without **e**, and with **f**, staining for MHC-I, whereas mononuclear cells in both tissues do stain for MHC-I (positive control).

### Discussion

The development of malignant tumours can be regarded as an example of cells escaping immune surveillance. One possible mechanism is down-regulation of MHC-I expression, affecting recognition and clearance by tumour specific CTLs. MHC-I alterations have been found in several human malignancies such as colon cancer (McDougall *et al.*, 1990), Burkitt lymphomas (Andersson *et al.*, 1991), small cell lung cancer (Doyle *et al.*, 1985), breast cancer (Fleming *et al.*, 1981) and melanomas (Brocker *et al.*, 1985). Also in cervical carcinomas this phenomenon has been observed in a substantial number of the carcinomas examined (Connor & Stern, 1990). However, the role of this phenomenon in the development of these malignancies is as yet unclear.

In this study formalin fixed, paraffin embedded material of

**Figure 2** MHC class II (MHC-II) expression in normal cervical epithelium **a**, CIN lesions **b-d**, and in cervical carcinomas **e**, **f**, as shown by MoAb LN3, specific for HLA-DR locus products. Size bars represent 50 μm. **a**, Normal squamous epithelial cells of the uterine cervix lack MHC-II. Weak staining of dendritic cells in the epithelial layer and mononuclear cells in the stroma. **b**, CIN III lesion. Most of the atypical cells do not stain for MHC-II, whereas mononuclear cells in the stroma do stain. **c**, CIN III lesion, showing heterogenous, diffuse staining for MHC-II on superficial atypical cells. Basal layers show no or very weak staining. **d**, CIN II lesion, in which cell surface expression of MHC-II on atypical epithelial cells can be seen. **e** and **f**, Cervical carcinomas showing no **e**, and strong **f**, staining for MHC-II, whereas mononuclear cells in the stroma do show MHC-II expression.

94 CIN lesions of different grades and 27 cervical carcinomas was analysed for MHC-I expression by immunohistochemistry using class I heavy chain specific antibodies HC10 and RaHC. In a substantial number of CIN lesions and carcinomas dysplastic cells showed a strongly reduced or even negative staining for MHC-I antigens, whereas normal epithelial cells and immunocompetent cells in the same sections were positive for MHC-I. This indicates that the observed alterations in MHC-I expression are related to dysplastic changes. This is also substantiated by the lack of MHC-I alterations in six normal cervical tissues of women undergoing hysterectomy for benign disease of the uterus such as leiomyoma and prolapse. Both the monoclonal HC10 antibody and polyclonal RaHC showed similar results, which indicates that not one epitope is affected, but rather the amount of intact wild type heavy chain polypeptides is

**Figure 3** Total HPV prevalence and HPV 16/18/31 prevalence in CIN lesions and squamous cell carcinomas (SCC). HPV DNA was detected and genotyped by a combined general primer-mediated and type specific PCR strategy.

**Table I** MHC class I (MHC-I) and II (MHC-II) expression patterns in CIN I to III lesions and squamous cell carcinomas (SCC)

| | | *Cervical lesion* | | | | |
|---|---|---|---|---|---|---|
| | | *Group 1* *CIN I (n = 34)* | *Group 2* *CIN II (n = 32)* | *Group 3* *CIN III (n = 28)* | *Group 4* *SCC (n = 27)*[a] | *P-value between* |
| MHC-I | normal | 11 | 7 | 4 | 5 | group 1 *vs* group 3: 0.09 |
| | disturbed | 11 | 10 | 12 | 13 | group 1 *vs* group 4: 0.21 |
| | heterogenous | 12 | 15 | 12 | 9 | group 1 *vs* group 4: 0.29 |
| MHC-II | normal | 22 | 12 | 11 | 10 | group 1 *vs* group 2: 0.03 |
| | altered | 12 | 20 | 17 | 14 | group 1 *vs* group 2: 0.03 |

The lowest *P*-values between the different groups are given for each MHC expression pattern. *P*-values below 0.01 ($P<0.01$) by Chi-square test is regarded to indicate a significant difference. [a]Only 24 samples were examined for MHC class II.

**Table II** MHC class I (MHC-I) and II (MHC-II) expression patterns for lesions, positive for HPV 16/18/31, positive for HPV 6/X and negative for any HPV type as determined by PCR

| | | *HPV status* | | | |
|---|---|---|---|---|---|
| | | *Group 1* *HPV 16/18/31 (n = 96)*[a] | *Group 2* *HPV 6/X (n = 11)* | *Group 3* *HPV neg. (n = 14)* | *P-value between* |
| MHC-I | normal | 18 | 3 | 6 | group 1 *vs* group 3: 0.04 |
| | disturbed | 37 | 5 | 4 | group 2 *vs* group 3: 0.38 |
| | heterogenous | 41 | 3 | 4 | group 1 *vs* group 3: 0.31 |
| MHC-II | normal | 40 | 6 | 9 | group 1 *vs* group 3: 0.14 |
| | altered | 53 | 5 | 5 | group 1 *vs* group 3: 0.14 |

The lowest *P*-values between the different groups are given for each MHC expression pattern. *P*-values below 0.01 ($P<0.01$) by Chi-square test is regarded to indicate a significant difference. [a]Only 93 samples were examined for MHC class II.

reduced or alternatively large modifications have taken place affecting the recognition by both antibodies.

The percentage of MHC-I downregulation (heterogenous and disturbed patterns) in carcinomas in our study (77%) is comparable with the percentage of complete and allele-specific down-regulation taken together in the carcinoma group studied by Connor & Stern (1990). However, when only analysing complete loss of MHC-I expression the percentage of carcinomas showing MHC-I alterations observed using HC10 and RaHC (48%) was considerably higher than obtained with W6/32 (9%) by Connor & Stern (1990). The monoclonal antibody W6/32 recognises MHC-I heavy chains, complexed with $\beta_2$-microglobulin, and is reactive only on frozen tissue, whereas HC10 and RaHC used in our study recognise MHC-I heavy chain antigens both in frozen and paraffin embedded tissue (Stam *et al.*, 1990). This implicates that the reduced staining for MHC-I heavy chains observed in our study cannot be caused by formalin fixation. One possible explanation for the different MHC-I staining patterns could be the presence of incomplete or modified heavy chains, which stain positive with W6/32, but lack the epitopes recognised by HC10/RaHC. Another possibility might be a difference in staining sensitivity between W6/32 and HC10/RaHC. In this case allele-specific down-regulation, leading to reduced steady state levels of MHC-I heavy chains, could result in the loss of staining with HC10/RaHC, while W6/32 staining is positive. Thus the negative staining with HC10 and RaHC in CIN lesions and cervical carcinomas, as observed in this study, can be based on total down-regulation of MHC-I as well as large modifications of the heavy chains. Also allele-specific down-regulation of MHC-I cannot be excluded.

**Figure 4** Immunohistochemical analysis of MHC-I expression and RNA *in situ* hybridisation (RISH) with an HPV 16 E7-specific probe, performed on consecutive tissue sections of a CIN II (panels **a**, **b**) and CIN I (panels **c**, **d**) lesion. **a**, Immunohistochemical staining of CIN II section using HC10 monoclonal antibody. Basal and parabasal cells are stained, with the signal localised on the cell membrane. **b**, Reflection signal of antisense HPV 16-E7 probe, obtained by confocal laserscan microscopy. Signal (white dots) is restricted to dysplastic epithelial cells, while stroma cells show background levels of signal. **c**, Detail of a CIN I lesion heterogenously stained with HC10 monoclonal antibody. **d**, RISH shows HPV 16-E7 transcripts in both dysplastic areas that show reduced staining for MHC-I and in areas that are positively stained for MHC-I. Size bars represent 30 $\mu$m.

Changes in MHC-II expression were detected as well in a substantial number of CIN lesions and cervical carcinomas. MHC-II upregulation on epithelial cells could enhance tumor-specific immunity by bypassing the classical antigen presenting cell (APC) mediated route and directly presenting antigen in the context of MHC-II to T-helper cells (Ostrand-Rosenberg *et al.*, 1991). This would result in a shorter and faster pathway of local lymphokine production, giving help to CTL-mediated killing and possibly also to other cells like natural killer cells. This could explain the favourable prognosis of tumours expressing MHC-II antigens (Esteban *et al.*, 1990; Gutierrez *et al.*, 1987). However, upregulation of MHC-II expression in melanoma is reported to be associated with a shorter disease-free survival (Ruiter *et al.*, 1986), indicating that de novo MHC-II expression on malignant cells does not always indicate a favourable prognosis. Upregulation of MHC-II has been reported in cervical carcinomas (Ferguson *et al.*, 1985; Glew *et al.*, 1992) and in CIN lesions (Warhol *et al.*, 1989). Our results show no significant differences in MHC-II expression between different groups of CIN and cervical carcionomas, suggesting that upregulated MHC-II expression does not supply help to a possible cytotoxic response against premalignant cervical lesions.

A number of lesions in this study showed alterations of both MHC classes, while other lesions exhibited normal MHC-I expression with altered MHC-II expression, and vice versa. Apparently, alteration of one MHC class is not exclusively correlated with alteration of the other. Thus different mechanisms may be involved in the regulation of MHC expression of both classes. It seems that a scale of different MHC-I and -II expression patterns can be observed in CIN lesions and cervical carcinomas indicating that local differences may exist in the cellular immune response to HPV induced premalignant and malignant cervical lesions.

Aberrant MHC-I expression could be observed in HPV negative lesions, as well as lesions containing high-risk HPVs (i.e. HPV 16, 18 and 31) and HPV types 6 or X, as determined by PCR. Normal MHC-I expression patterns were also found in all these groups. This indicates that variations of MHC-I expression are not directly correlated with the presence of specific HPV genotypes. Also no correlation could be found between MHC-II expression and presence of any of the HPV types either, which suggests that the process of upregulation of MHC-II is not related to the presence of HPV DNA. This is in agreement with the observation on cervical carcinomas by Connor & Stern (1990) and Glew *et al.* (1992), using Southern blot analysis for HPV typing. Since the PCR, performed on cell extracts, cannot supply morphological information on HPV distribution additional *in situ* hybridisation was carried out to establish that changes in MHC expression and HPV presence were localised in the same dysplastic/neoplastic cells. For optimal sensitivity of HPV detection RNA instead of DNA *in situ* hybridisation was performed, because with the latter method low copy numbers of HPV genotypes might not be detected (Walboomers *et al.*, 1988). The HPV 16-E7 RNA probe was used for this purpose since this gene is highly expressed in HPV transformed cells and E7 transcripts can be detected in all neoplastic cells of HPV 16 containing cervical carcinomas (van den Brule *et al.*, 1991*a*). Results showed that E7 transcripts were found in cells with normal as well as in cells with changes in MHC expression in CIN lesions and carcinomas. This indicates that presence of E7 transcripts alone does not result in altered MHC expression. Further transcriptional analysis is necessary to reveal whether other HPV encoded proteins play a role in aberrant expression of MHC-I or -II. Since the expression of several early and all late HPV genes can be influenced by HPV DNA integration, also the HPV physical state could be an important factor and needs to be further analysed.

Recently we have investigated HPV 16 E7 transcriptionally active cervical carcinomas (van den Brule *et al.*, 1991*a*), which show a disturbed MHC-I expression using RISH with heavy chain locus-specific antisense RNA probes. All carcinomas, that show MHC-I downregulation as detected with HC10, showed abundant MHC-I heavy chain transcripts in carcinoma cells. This indicates that down-regulation occurs at the post-transcriptional level (Walboomers *et al.*, abstract PV workshop Seattle 1991, manuscript in preparation). Similar studies have to be performed for MHC-II expression using RISH, to establish if upregulated MHC-II expression occurs at the transcriptional or post-transcriptional level.

Finally, it has also to be taken into consideration that the biological behaviour of CIN lesions is very heterogenous and cannot be predicted by a single histological observation. In general it is assumed that only a minority of CIN lesions show progression to invasive cancer (Richart, 1987). This means that from randomly taken CIN lesions only a small percentage (approximately 10%) represent progressive CIN. Therefore it seems important to define the clinical parameters of the study groups when MHC expression levels in CIN lesions are compared with results obtained by others. CIN lesions analysed in this study originated from a selected group of women attending the oncological gynaecological clinic, having a high recurrency rate of CIN and are more prone to develop cervical cancer. Currently, this study is extended with CIN lesions of patients that could be retrospectively (Gaarenstroom *et al.*, submitted) and prospectively defined on clinical behaviour. Patients, exhibiting regressive, progressive or persistent CIN disease will be analysed for MHC expression and presence of HPV genotypes. These data will give information about the biological significance of disturbed MHC-I expression, as detected by HC10 and RaHC, as well as W6/32.

The authors would like to thank Mss Mireille de Wit and Danielle Wolvers for technical assistance, and Dr H.L. Ploegh from the Netherlands Cancer Institute, Amsterdam, The Netherlands, for supplying antibodies RaHC and HC10. This work was supported by grants from the Prevention Fund (28-1502.2) and the Dutch Cancer Society 'Koningin Wilhelmina Fonds' (IKA VU 91-10), The Netherlands. Dr P.L. Stern was supported by the Cancer Research Campaign of Great Britain.

### References

ANDERSSON, M.L., STAM, N.J., KLEIN, G., PLOEGH, H.L. & MASUCCI, M.G. (1991). Aberrant expression of HLA class I antigens in Burkitt Lymphomas cells. *Int. J. Cancer*, **47**, 544–549.

BROCKER, E., SUTER, L., BRUGGEN, J., RUITER, D.J., MACHER, E. & SORG, C. (1985). Phenotypic dynamics of tumor progression in human malignant melanoma. *Int. J. Cancer*, **41**, 29–35.

CONNOR, M.E. & STERN, P.L. (1990). Loss of MHC class-I expression in cervical carcinomas. *Int. J. Cancer*, **46**, 1029–1035.

DOYLE, A., MARTIN, A., FUNA, K., GAZDAR, A., CARNEY, D., MARTIN, S., LINNOILA, I., CUTTUTTA, F., MULSHINE, J.J., BUNN, P. & MINNA, J. (1985). Markedly decreased expression of class I histocompatibility antigens, protein, and mRNA in human small-cell lung cancer. *J. Exp. Med.*, **161**, 1135–1139.

DÜRST, M., DZARLIEVA-PETRUSEVSKA, T., BOUKAMP, P., FUSENIG, N.E. & GISSMANN, L. (1987). Molecular and cytogenetic analysis of immortalized human primary keratinocytes obtained after transfection with human papillomavirus type 16 DNA. *Oncogene*, **1**, 251–256.

ESTEBAN, F., RUIZ-CABELLO, F., CONCHAS, A., PEREZ-AYALA, M., SANCHEZ-ROSAS, J.A. & GARRIDO, F. (1990). HLA-DR expression is associated with excellent prognosis in squamous cell carcinomas of the larynx. *Clin. Exp. Metastases*, **8**, 319–328.

FERGUSON, A., MOORE, M. & FOX, H. (1985). Expression of MHC products and leucocyte differentiation antigens in gynaecological neoplasms: an immunohistological analysis of the tumour cells and infiltrating lymphocytes. *Br. J. Cancer*, **52**, 551–557.

FLEMING, K., MCMICHAEL, A., MORTON, J., WOODS, J. & MCGEE, J. (1981). Distribution of HLA class I antigens in normal human tissue and in mammary cancer. *J. Clin. Pathol.*, **34**, 779–785.

GAARENSTROOM, K.N., MELKERT, P., WALBOOMERS, J.M.M., VAN BOMMEL, P.F.J., HOPMAN, E., MEIJER, C.J.L.M., KENEMANS, P., VAN DEN BRULE, A.J.C. & HELMERHORST, Th.J.M. (1992). The presence of oncogenic HPV types is strongly related with progression of CIN lesions: results of a retrospective study. *Submitted.*

GLEW, S.S., DUGGAN-KEEN, M., CABRERA, T. & STERN, P.L. (1992). HLA class II antigens in human papillomavirus-associated cervical cancer. *Cancer Res.*, **52**, 4009–4015.

GUTIERREZ, J., LOPEZ-NEVOT, M.A., CABRERA, T., OLIVA, M.R., ESQUIVAS, J., RUIZ-CABELLO, F. & GARRIDO, F. (1987). Class I and II antigen distribution in normal mucosa, adenoma and colon carcinoma: relation with malignancy and invasiveness. *Exp. Clin. Immunogenet*, **4**, 144–152.

LUNGU, O.L., SUN, X.W., FELIX, J., RICHART, R.M., SILVERSTEIN, S. & WRIGHT, T.C. (1992). Relationship of human papillomavirus type to grade of cervical intraepithelial neoplasia. *J.A.M.A*, **267**, 2493–2496.

MCCANCE, D., KOPAN, R., FUCHS, E. & LAIMINS, L.A. (1988). Human papillomavirus type 16 alters human epithelial differentiation in vitro. *Proc. Natl. Acad. Sci. USA*, **85**, 7169–7173.

McDOUGALL, C.J., NGOI, S.S., GOLDMAN, I.S., GODWIN, T., FELIX, J., DECOSSE, J.J. & RIGAS, B. (1990). Reduced expression of HLA class I and II antigens in colon cancer. *Cancer Res.*, **50**, 8023–1027.

OUDEJANS, C.B.M., KRIMPENFORT, P., PLOEGH, H.L. & MEIJER, C.J.L.M. (1989). Lack of expression of HLA-B27 gene in transgenic mouse trophoblast. *J. Exp. Med.*, **169**, 447–456.

OSTRAND-ROSENBERG, S., ROBY, C., CLEMENTS, V.K. & COLE, G.A. (1991). Tumor-specific immunity can be enhanced by transfection of tumor cells with syngeneic MHC class II genes or allogeneic MHC class I genes. *Int. J. Cancer*, (suppl.) **6**, 61–68.

PIRISI, L., YASUMOTO, S., FELLER, M., DONIGER, J. & DIPAOLO, J.A. (1987). Transformation of human fibroblasts and keratinocytes with human papillomavirus type 16 DNA. *J. Virol.*, **61**, 1061–1066.

RICHART, R.M. (1987). Causes and management of cervical intraepithelial neoplasia. *Cancer*, **60**, 1951–1959.

RUITER, D.J., BROCKER, E.B. & FERRONE, S. (1986). Expression and susceptability to modulation by interferons of HLA class I and II antigens in melanoma cells. Immunohistochemical analysis and clinical relevance. *J. Immunogenetics*, **13**, 229–234.

RUIZ-CABELLO, F., KLEIN, E. & GARRIDO, F. (1991). MHC antigens on human tumors. *Immunology letters*, **29**, 181–190.

SCHRIER, P.I., BERNARDS, R., VAESSEN, R.T.M.J., HOUWELING, A. & VAN DER EB, A.J. (1983). Expression of class I Major Histocompatibility antigens is switched off by highly oncogenic adenovirus 12 in transformed rat cells. *Nature*, **305**, 771–775.

SIGNAS, C., KAFZE, A., PERSSON, H. & PHILIPSON, L. (1982). An adenovirus glycoprotein binds heavy chains of class I transplantation antigens from man and mouse. *Nature*, **299**, 175–178.

SNIJDERS, P.J.F., VAN DEN BRULE, A.J.C., SCHRIJNEMAKERS, H.F.J., SNOW, G., MEIJER, C.J.L.M. & WALBOOMERS, J.M.M. (1990). The use of general primers in the polymerase chain reaction permits the detection of a broad spectrum of human papillomavirus genotypes. *J. Gen. Virol.*, **71**, 173–181.

STAM, N.J., SPITS, H. & PLOEGH, H.L. (1986). Monoclonal antibodies raised against denatured HLA-B locus heavy chains permit biochemical characterisation of certain HLA-C locus products. *J. Immunol.*, **137**, 2299–2306.

STAM, N.J., VROOM, Th.M., PETERS, P.J., PASTOORS, E.B. & PLOEGH, H.L. (1990). HLA-A and HL-B-specific monoclonal antibodies reactive with free heavy chains in Western blots, in formalin fixed, paraffin embedded tissue sections and in cryo-immunoelectron microscopy. *Int. Immunol.*, **2(2)**, 113–125.

VAN DEN BRULE, A.J.C., MEIJER, C.J.L.M., BAKELS, V., KENEMANS, P. & WALBOOMERS, J.M.M. (1990). Rapid Human Papillomavirus detection in cervical scrapes by combined general primer-mediated and type-specific polymerase chain reaction. *J. Clin. Microbiol.*, **28**, 2739–2743.

VAN DEN BRULE, A.J.C., WALBOOMERS, J.M.M., DU MAINE, M., KENEMANS, P. & MEIJER, C.J.L.M. (1991). Difference in prevalence of human papillomavirus genotypes in cytomorphologically normal cervical smears is associated with a history of cervical intraepithelial neoplasia. *Int. J. Cancer*, **48**, 404–408.

VAN DEN BRULE, A.J.C., CROMME, F.V., SNIJDERS, P.J.F., SMIT, L., OUDEJANS, C.B.M., BAAK, J.P.A., MEIJER, C.J.L.M. & WALBOOMERS, J.M.M. (1991*a*). Non radioactive RNA *in situ* hybridisation detection of HPV-16 E7 transcripts in squamous cell carcinomas of the uterine cervix using confocal laserscan microscopy. *Am. J. Pathol*, **139(5)**, 1037–1045.

WALBOOMERS, J.M.M., MELCHERS, W.J.G., MULLINK, H., MEIJER, C.J.L.M., STRUYK, A., QUINT, W.G.J., VAN DEN NOORDA, J. & TER SCHEGGET, J. (1988). Sensitivity of in situ detection with biotinylated probes of human papillomavirus type 16 DNA in frozen tissue sections of squamous cell carcinomas of the cervix. *Am. J. Pathol.*, **131**, 587–594.

WALBOOMERS, J.M.M., MELKERT, P.W.J., VAN DEN BRULE, A.J.C., SNIJDERS, P.J.F. & MEIJER, C.J.L.M. (1992). The polymerase chain reaction for screening in diagnostic cytopathology of the cervix. In: *Diagnostic Molecular Pathology* vol. 2, Herrington, C.S. & McGee, O.D. (eds), p. 153–172. IRL press, Oxford, UK.

WARHOL, M.J. & McGEE, B. (1989). The expression of histocompatibility antigen (HLA-DR in cervical squamous epithelium infected with human papillomavirus. *Mod. Pathol.*, **2(2)**, 101–106.

WOODWORTH, C.D., WAGGONER, S., BARNES, W., STOLER, M.H. & DIPAOLO, J.A. (1990). Human cervical and foreskin epithelial cells immortalized by human papillomavirus DNAs exhibit dysplastic differentiation in vitro. *Cancer Res.*, **50**, 3709–3715.

ZUR HAUSEN, H. (1989). Papillomavirus in anogenital cancer: the dilemma of epidemiologic approaches. *J. Natl. Cancer Inst.*, **81**, 1680–1682.

ZUR HAUSEN, H. (1989*a*). Papillomavirus in anogenital cancer as a model to understand the role of viruses in human cancers. *Cancer Res.*, **449**, 4677–4681.


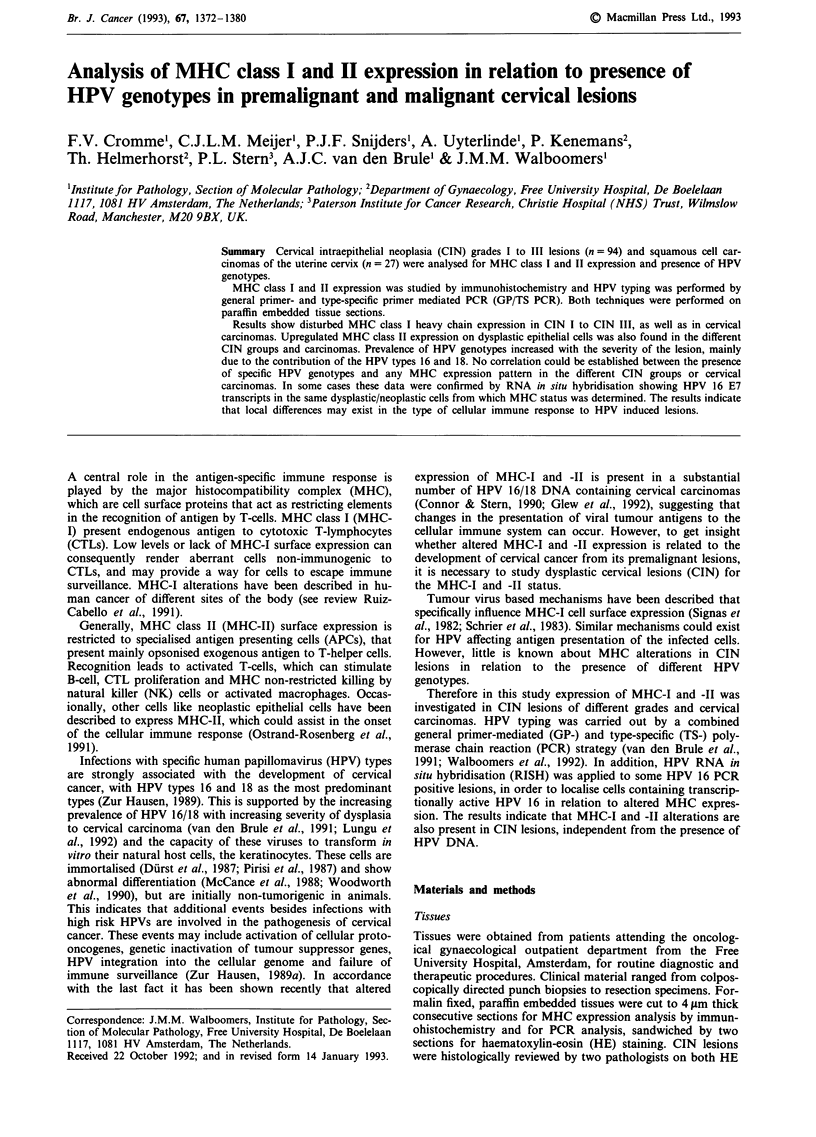

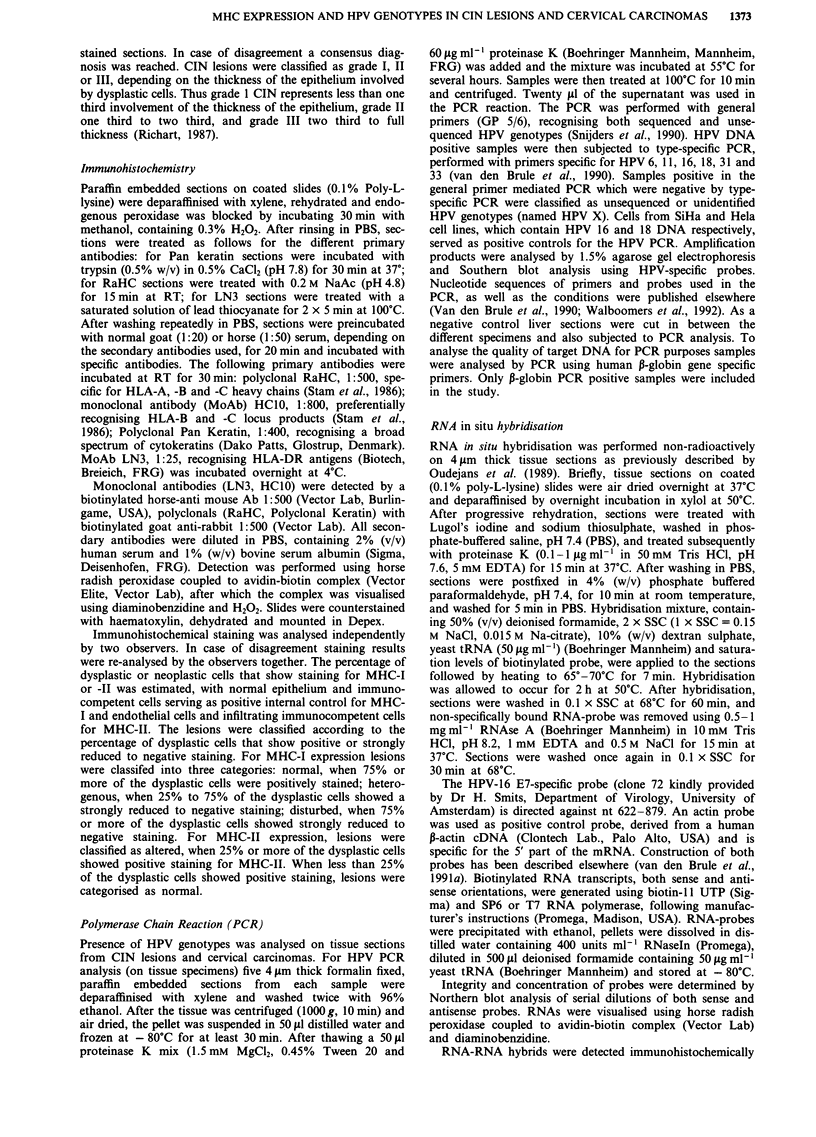

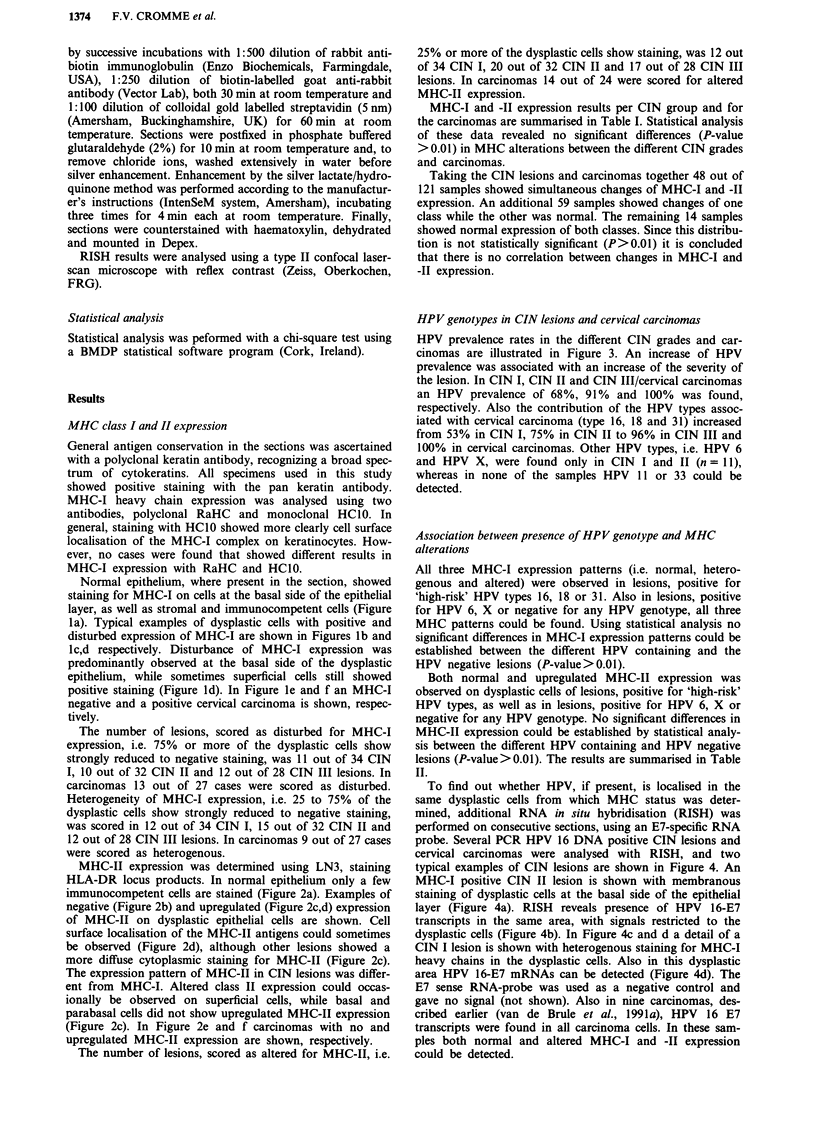

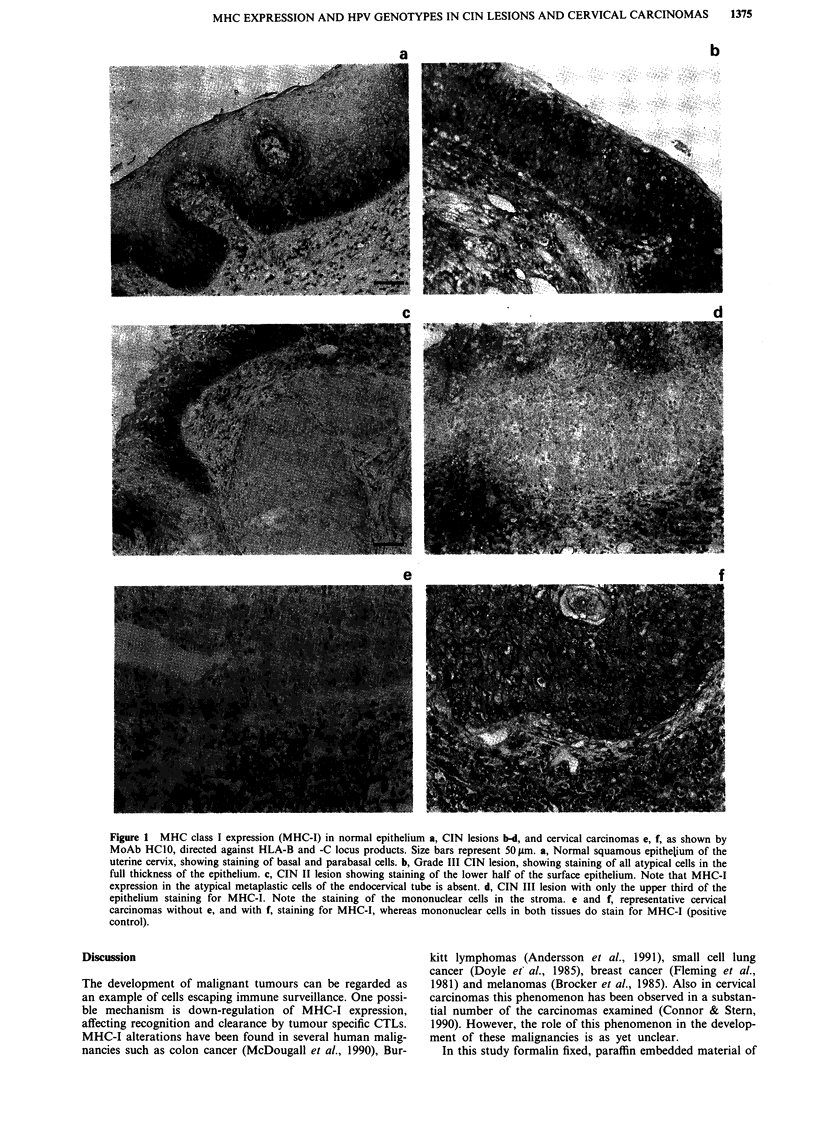

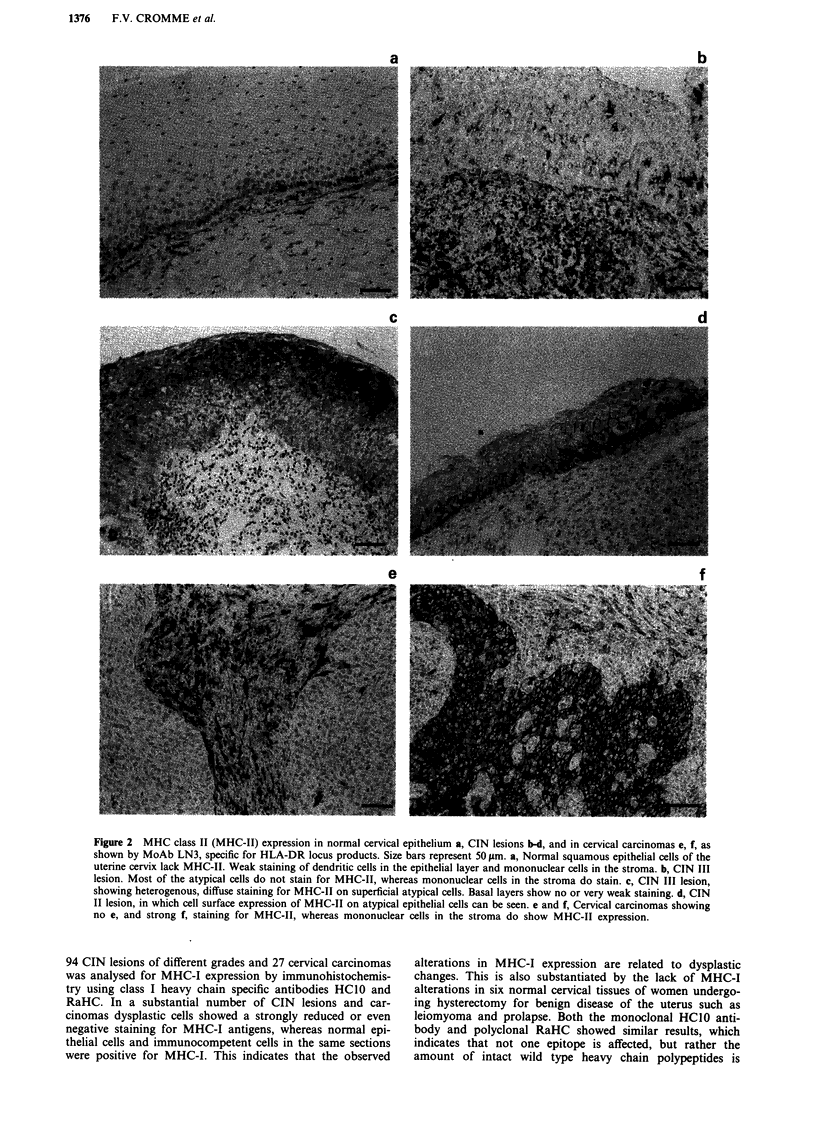

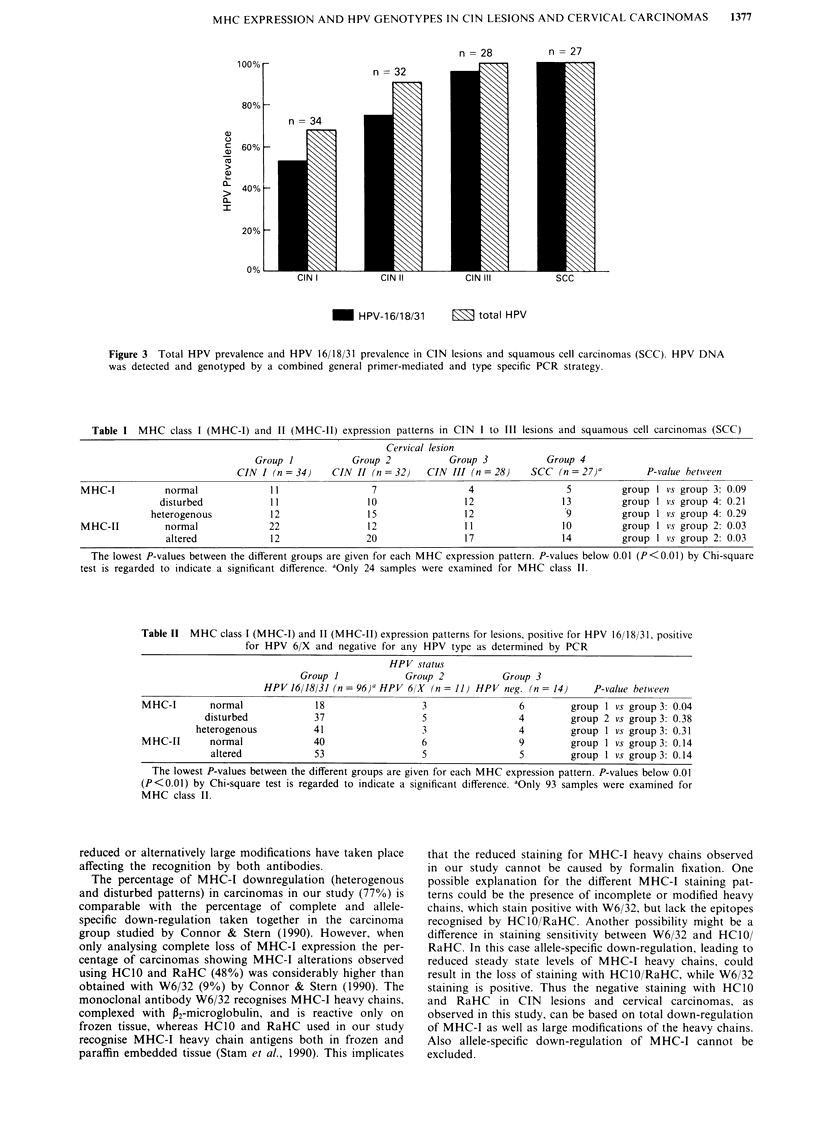

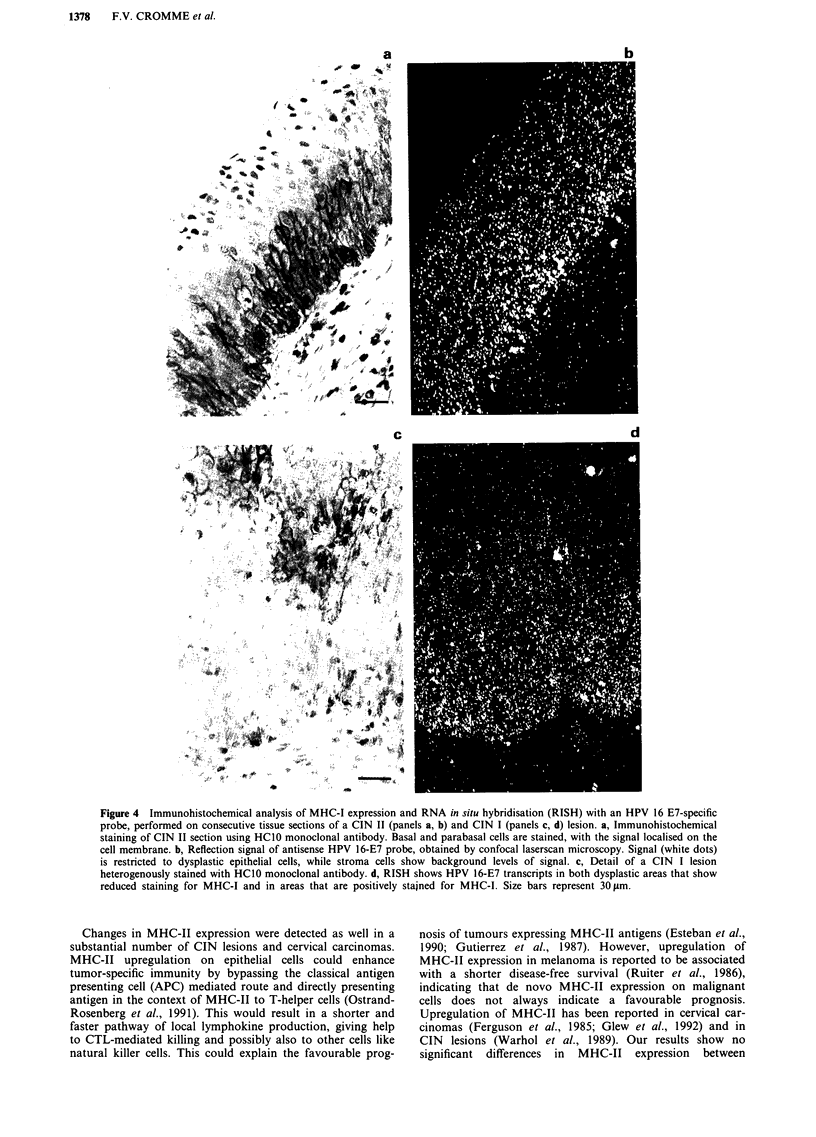

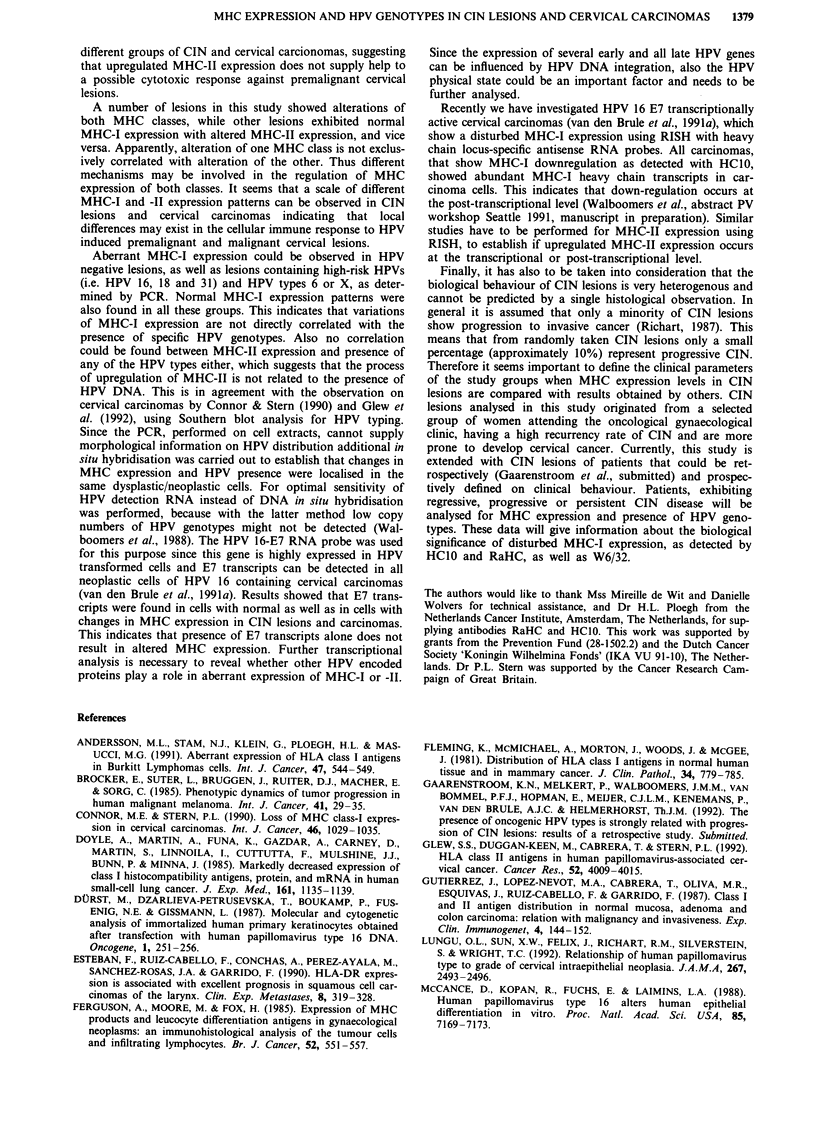

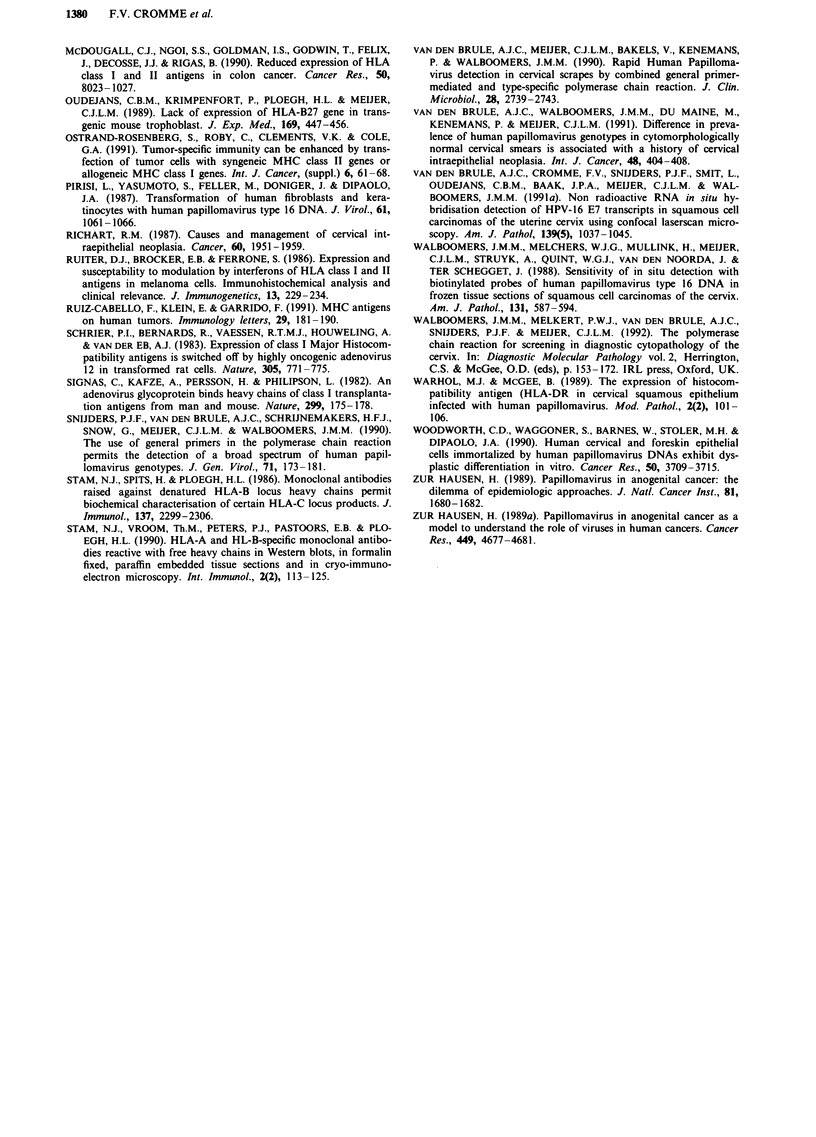


## References

[OCR_00828] Andersson M. L., Stam N. J., Klein G., Ploegh H. L., Masucci M. G. (1991). Aberrant expression of HLA class-I antigens in Burkitt lymphoma cells.. Int J Cancer.

[OCR_00831] Bröcker E. B., Suter L., Brüggen J., Ruiter D. J., Macher E., Sorg C. (1985). Phenotypic dynamics of tumor progression in human malignant melanoma.. Int J Cancer.

[OCR_00836] Connor M. E., Stern P. L. (1990). Loss of MHC class-I expression in cervical carcinomas.. Int J Cancer.

[OCR_00840] Doyle A., Martin W. J., Funa K., Gazdar A., Carney D., Martin S. E., Linnoila I., Cuttitta F., Mulshine J., Bunn P. (1985). Markedly decreased expression of class I histocompatibility antigens, protein, and mRNA in human small-cell lung cancer.. J Exp Med.

[OCR_00849] Dürst M., Dzarlieva-Petrusevska R. T., Boukamp P., Fusenig N. E., Gissmann L. (1987). Molecular and cytogenetic analysis of immortalized human primary keratinocytes obtained after transfection with human papillomavirus type 16 DNA.. Oncogene.

[OCR_00854] Esteban F., Ruiz-Cabello F., Concha A., Pérez-Ayala M., Sánchez-Rozas J. A., Garrido F. (1990). HLA-DR expression is associated with excellent prognosis in squamous cell carcinoma of the larynx.. Clin Exp Metastasis.

[OCR_00860] Ferguson A., Moore M., Fox H. (1985). Expression of MHC products and leucocyte differentiation antigens in gynaecological neoplasms: an immunohistological analysis of the tumour cells and infiltrating leucocytes.. Br J Cancer.

[OCR_00866] Fleming K. A., McMichael A., Morton J. A., Woods J., McGee J. O. (1981). Distribution of HLA class 1 antigens in normal human tissue and in mammary cancer.. J Clin Pathol.

[OCR_00877] Glew S. S., Duggan-Keen M., Cabrera T., Stern P. L. (1992). HLA class II antigen expression in human papillomavirus-associated cervical cancer.. Cancer Res.

[OCR_00882] Gutierrez J., López-Nevot M. A., Cabrera T., Oliva R., Esquivias J., Ruiz-Cabello F., Garrido F. (1987). Class I and II HLA antigen distribution in normal mucosa, adenoma and colon carcinoma: relation with malignancy and invasiveness.. Exp Clin Immunogenet.

[OCR_00889] Lungu O., Sun X. W., Felix J., Richart R. M., Silverstein S., Wright T. C. (1992). Relationship of human papillomavirus type to grade of cervical intraepithelial neoplasia.. JAMA.

[OCR_00895] McCance D. J., Kopan R., Fuchs E., Laimins L. A. (1988). Human papillomavirus type 16 alters human epithelial cell differentiation in vitro.. Proc Natl Acad Sci U S A.

[OCR_00903] McDougall C. J., Ngoi S. S., Goldman I. S., Godwin T., Felix J., DeCosse J. J., Rigas B. (1990). Reduced expression of HLA class I and II antigens in colon cancer.. Cancer Res.

[OCR_00914] Ostrand-Rosenberg S., Roby C., Clements V. K., Cole G. A. (1991). Tumor-specific immunity can be enhanced by transfection of tumor cells with syngeneic MHC-class-II genes or allogeneic MHC-class-I genes.. Int J Cancer Suppl.

[OCR_00909] Oudejans C. B., Krimpenfort P., Ploegh H. L., Meijer C. J. (1989). Lack of expression of HLA-B27 gene in transgenic mouse trophoblast. Conserved genetic pressures underlying extra-embryonic development.. J Exp Med.

[OCR_00919] Pirisi L., Yasumoto S., Feller M., Doniger J., DiPaolo J. A. (1987). Transformation of human fibroblasts and keratinocytes with human papillomavirus type 16 DNA.. J Virol.

[OCR_00925] Richart R. M. (1987). Causes and management of cervical intraepithelial neoplasia.. Cancer.

[OCR_00929] Ruiter D. J., Bröcker E. B., Ferrone S. (1986). Expression and susceptibility to modulation by interferons of HLA class I and II antigens on melanoma cells. Immunohistochemical analysis and clinical relevance.. J Immunogenet.

[OCR_00935] Ruiz-Cabello F., Klein E., Garrido F. (1991). MHC antigens on human tumors.. Immunol Lett.

[OCR_00939] Schrier P. I., Bernards R., Vaessen R. T., Houweling A., van der Eb A. J. Expression of class I major histocompatibility antigens switched off by highly oncogenic adenovirus 12 in transformed rat cells.. Nature.

[OCR_00945] Signäs C., Katze M. G., Persson H., Philipson L. (1982). An adenovirus glycoprotein binds heavy chains of class I transplantation antigens from man and mouse.. Nature.

[OCR_00950] Snijders P. J., van den Brule A. J., Schrijnemakers H. F., Snow G., Meijer C. J., Walboomers J. M. (1990). The use of general primers in the polymerase chain reaction permits the detection of a broad spectrum of human papillomavirus genotypes.. J Gen Virol.

[OCR_00957] Stam N. J., Spits H., Ploegh H. L. (1986). Monoclonal antibodies raised against denatured HLA-B locus heavy chains permit biochemical characterization of certain HLA-C locus products.. J Immunol.

[OCR_00965] Stam N. J., Vroom T. M., Peters P. J., Pastoors E. B., Ploegh H. L. (1990). HLA-A- and HLA-B-specific monoclonal antibodies reactive with free heavy chains in western blots, in formalin-fixed, paraffin-embedded tissue sections and in cryo-immuno-electron microscopy.. Int Immunol.

[OCR_00977] Van Den Brule A. J., Walboomers J. M., Du Maine M., Kenemans P., Meijer C. J. (1991). Difference in prevalence of human papillomavirus genotypes in cytomorphologically normal cervical smears is associated with a history of cervical intraepithelial neoplasia.. Int J Cancer.

[OCR_00992] Walboomers J. M., Melchers W. J., Mullink H., Meijer C. J., Struyk A., Quint W. G., van der Noordaa J., ter Schegget J. (1988). Sensitivity of in situ detection with biotinylated probes of human papilloma virus type 16 DNA in frozen tissue sections of squamous cell carcinomas of the cervix.. Am J Pathol.

[OCR_01006] Warhol M. J., Gee B. (1989). The expression of histocompatibility antigen HLA-DR in cervical squamous epithelium infected with human papilloma virus.. Mod Pathol.

[OCR_01012] Woodworth C. D., Waggoner S., Barnes W., Stoler M. H., DiPaolo J. A. (1990). Human cervical and foreskin epithelial cells immortalized by human papillomavirus DNAs exhibit dysplastic differentiation in vivo.. Cancer Res.

[OCR_00987] van den Brule A. J., Cromme F. V., Snijders P. J., Smit L., Oudejans C. B., Baak J. P., Meijer C. J., Walboomers J. M. (1991). Nonradioactive RNA in situ hybridization detection of human papillomavirus 16-E7 transcripts in squamous cell carcinomas of the uterine cervix using confocal laser scan microscopy.. Am J Pathol.

[OCR_00970] van den Brule A. J., Meijer C. J., Bakels V., Kenemans P., Walboomers J. M. (1990). Rapid detection of human papillomavirus in cervical scrapes by combined general primer-mediated and type-specific polymerase chain reaction.. J Clin Microbiol.

[OCR_01018] zur Hausen H. (1989). Papillomavirus in anogenital cancer: the dilemma of epidemiologic approaches.. J Natl Cancer Inst.

